# Acute Pancreatitis Causing Descending Colonic Stricture: A Rare Sequelae

**DOI:** 10.1177/2324709619834594

**Published:** 2019-03-28

**Authors:** Amrendra Kumar Mandal, Paritosh Kafle, Pradip Puri, Baikuntha Chaulagai, Muhammad Hassan, Bikash Bhattarai, Rajan Kanth, Vijay Gayam

**Affiliations:** 1 Interfaith Medical Center, Brooklyn, NY, USA; 2 Carilion Clinic, Roanoke, VA, USA

**Keywords:** acute pancreatitis, colon stricture, Crohn’s disease

## Abstract

An isolated descending colonic stricture is an unlikely complication of acute pancreatitis, with the nonspecific symptoms of colonic stricture making the overall diagnosis difficult. Crohn’s disease (CD) and tuberculosis (TB) are the two common etiologies of an isolated colonic stricture and may present similarly to colonic stricture related to acute pancreatitis. Unfortunately, colonoscopy and biopsy often cannot determine the etiology, and surgical resection may be needed to provide both symptomatic relief and confirm the diagnosis. As a result, descending colonic stricture may produce a diagnostic dilemma with CD and TB as all 3 conditions may be radiologically and endoscopically indistinguishable. We describe a young male with weight loss and abdominal pain. TB testing was negative, with radiography and ELISA (enzyme-linked immunosorbent assay) testing supporting a diagnosis of the CD. The patient was initiated on sulfasalazine but worsened over the next month. Further investigations revealed that the patient had descending colonic stricture without CD. Therefore, the stricture’s etiology was most likely related to an episode of acute pancreatitis the patient had 2 months before admission and was found to have left colonic segment adherent to the pancreas eventually requiring segmentectomy. Although the pathophysiology of colonic stricture after pancreatitis is unclear, we speculate that inflammatory injury to the colon is an important component. Finally, we emphasize that colonic stricture is a rare complication of pancreatitis.

## Introduction

Large bowel obstruction is a relatively common condition, although it is a rare sequelae to acute or chronic pancreatitis and is associated with high mortality rates.^1-3^ To the best of our knowledge, only 2 cases of descending colon stricture secondary to pancreatitis have been reported in the literature.^4,5^ The diagnosis is formulated on a combination of barium enema, colonoscopy, and computed tomography (CT) imaging of the abdomen. The anatomic relationship of the large bowel to the pancreas is a vital factor in the genesis and localization of these lesions. Strictures related to pancreatitis may occur after a significant delay following the resolution of pancreatitis.^6^ Benign colorectal strictures can develop after diverticulitis, ischemic colitis, radiation colitis, or colonic resection. Large bowel necrosis represents an early complication of acute pancreatitis, with mortality rates between 50% and 90%.^7^

## Case Presentation

**Figure 1.**
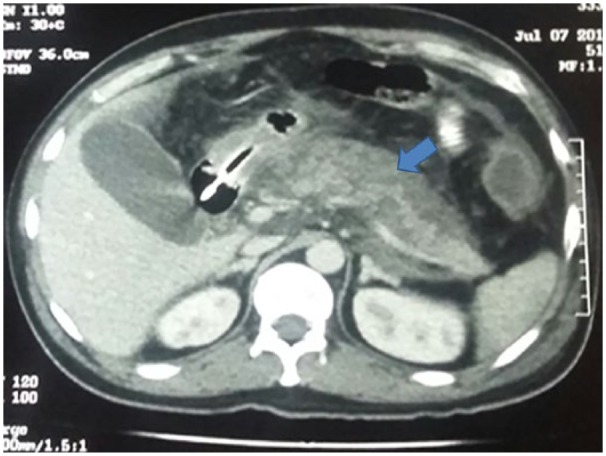
Abdominal computed tomography scan from the episode of acute pancreatitis 2 months ago showed necrotizing acute pancreatitis (blue arrow).

A 32-year-old man presented with colicky abdominal pain in the left lower quadrant for 2 weeks duration. He had a significant weight loss of approximately 30 kg over 2 months associated with decreased appetite. He also has a history of chronic alcoholism and smoking. There was no associated nausea, vomiting, hematemesis, melena, or hematochezia. Review of symptoms was negative for fever, arthritis, skin rash, jaundice, and pruritus. Two months prior, the patient was discharged from another hospital following a 6-week hospitalization for alcohol-induced necrotizing acute pancreatitis, which was treated successfully by conservative measures. Admission vitals revealed an afebrile and normotensive patient with a heart rate of 92 beats per minute and oxygen saturation of 98% on room air. On physical examination, the abdomen was soft with mild tenderness to deep palpation in the left iliac fossa and lumbar region. There were no signs of peritonitis. Laboratory results and abdominal sonogram showed no significant findings. Abdominal CT scan from the episode of acute pancreatitis 2 months ago showed findings consistent with necrotizing acute pancreatitis (Figure 1).

**Figure 2.**
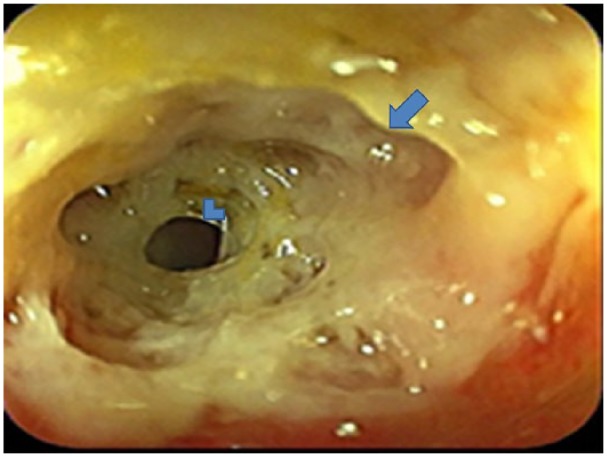
The colonoscope revealed ulceration with a partial stricture at the descending colon approximately 30 cm from the anal verge (blue arrow and arrow head showing distal and proximal stricture, respectively).

A colonoscopy was done because of the abdominal pain and significant weight loss. The scope revealed ulceration with a partial stricture at the descending colon approximately 30 cm from the anal verge (Figure 2). However, the scope could not be navigated beyond the stricture. Controlled radial expansion balloon dilatation was tried without success in dilating the stricture.

**Figure 3.**
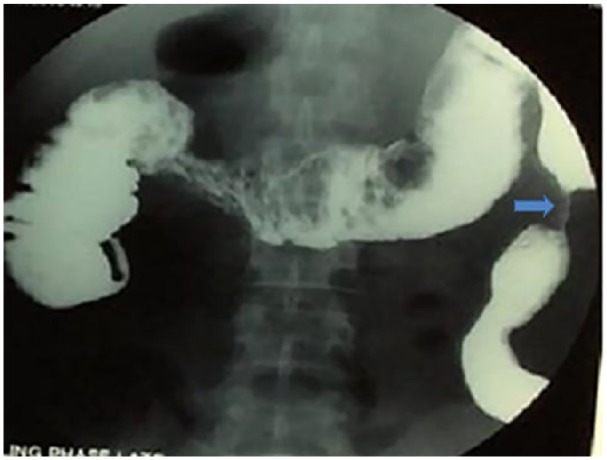
Gastrograffin enema study showing descending colonic stricture.

**Figure 4.**
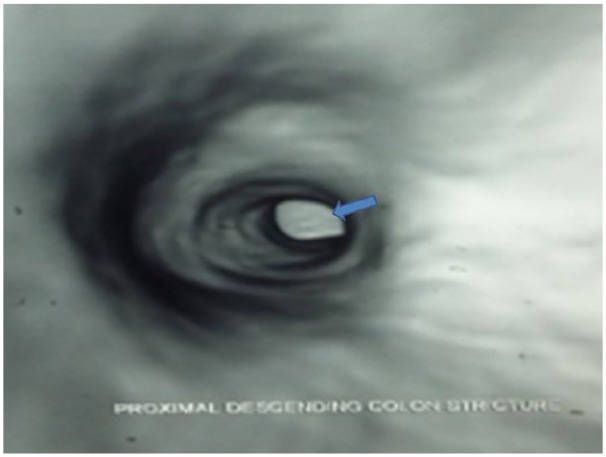
Computed tomography colonoscopy revealed descending colon stricture of approximately 6 cm in length.

Due to tuberculosis (TB) being a possible differential diagnosis, a workup for TB was warranted. This included sputum for acid-fast bacillus, chest X-ray, purified protein derivative test, erythrocyte sedimentation rate, and pathological examination, all of which were negative for TB. Biopsy of the lesion revealed active focal colitis with ulceration, but no granulomas were seen. Barium enema and CT colonoscopy were done to assess the length of the stricture to plan an appropriate treatment strategy as shown in Figures 3 and 4, respectively. Barium enema findings were consistent with inflammatory bowel disease, as a benign stricture was seen with no evidence of malignancy.

Based on the clinical presentation and barium enema, Crohn’s disease was high in the differential diagnosis. ASCA immunoglobulin G antibodies to saccharomyces cerevisiae was ordered with a result of 22.2 U/mL (>10 U/mL; ELISA [enzyme-linked immune sorbent assay]). The patient was thought to have had a CD at this point and was subsequently initiated on sulfasalazine. Despite treatment for CD, the abdominal pain persisted over the next month with worsening weight loss. Repeat colonoscopy was performed, which revealed further strictures requiring a segmentectomy with single layer extra mucosal end-to-end anastomosis. Anatomically, the left colonic segment was observed to be adherent to the pancreas. A surgical biopsy specimen revealed active focal colitis, and negative for TB and CD. A final diagnosis of colonic stricture due to acute necrotizing pancreatitis was made. Abdominal pain decreased postoperatively, and the patient was discharged on postoperative day 7. A follow-up colonoscopy 2 months later revealed healed anastomotic sites.

## Discussion

Stricture of the colon at the splenic flexure as a sequel to pancreatitis was first reported by Forlini et al in 1922.^8^ However, stricture of the descending colon is exceptionally rare. A case report by Yoo et al showed an obstruction of the descending colon as a complication of acute pancreatitis, and the patient underwent surgery due to nonresolving stricture.^4^ Another case of descending colon stricture presented as an acute intestinal obstruction secondary to acute pancreatitis and during the subsequent laparotomy; a tumor of the left colonic flexure with a fistula to a retroperitoneal abscess and necrosis of the pancreatic tail was observed.^5^ In a case series analyzing strictures formed during pancreatitis including a total of 13 colonic strictures, 9 strictures were located at the splenic flexure; 2 were adjacent to the splenic flexure; and 1 was at the hepatic flexure.^9^ Another retrospective case series involving 296 patients with acute pancreatitis showed that only 6.1% of patients developed colonic complications. Of these, only 1 case had incomplete colonic stenosis, and no strictures being described as occurring in the descending colon.^10^ This makes our patient an atypical presentation, as none of the strictures noted in both of the pancreatitis case series occurring at the level of the descending colon.

Since pancreatitis rarely manifests into a descending colonic stricture, and because of the weight loss, imaging, and ELISA results being consistent with CD, we presumed our patient to have CD. Up to 20% of patients with CD may present with isolated colitis. The clinical presentation of the colonic CD is highly variable between patients. This was part of the initial diagnostic dilemma in our case and the initiation of sulfasalazine.^11^

Colonic obstruction can arise during both the acute stage of pancreatitis and also a few weeks after the resolution of acute pancreatitis.^2,12^ Many pathological hypotheses have been suggested for the development of colonic obstruction following pancreatitis; acutely as a result of ischemia and necrosis due to acute pancreatitis.^10,13^ Additionally, the peritoneal reflections from the anterior surface of the pancreas provide a route for the spread of both pancreatic enzymes and inflammatory mediators within the transverse mesocolon and small bowel mesentery.^1^ This proximity of splenic flexure region to the pancreas explains the localization of these lesions, with the splenic flexure and transverse colon being the common sites for stenosis. Additionally, the splenic flexure is a watershed region between the areas of supply of the middle and left colic arteries and is particularly susceptible to periods of hypotension during acute pancreatitis, leading to an ischemic process leading to necrosis followed by stricture.^6^ Through-the-scope balloons may be used to reach and pneumatically dilate strictures.^14^ Through-the scope balloons have been used as a first-line treatment modality for benign colorectal strictures, but the success rate is variable in the literature.^15,16^ Predictors of a successful outcome include relatively narrow stenosis (<10 mm), a short segment stricture (<4 cm), and anastomotic strictures. Poor predictors of success include multiple strictures, complete obstruction, associated fistulas within the stricture, active inflammation around the stricture, recent surgery, a tight angulation, and malignancy.^16^ In our case, failure to dilate the stricture was likely due to more extensive involvement and a more severely stenosed segment. Postoperative histology provides valuable information as it can exclude primary large bowel pathologies such as inflammatory bowel disease including CD and neoplasia. In this case, histology confirmed the presence of scarring and peri-colonic inflammatory changes ruling out CD as well as other etiologies.

## Conclusion

Clinicians should consider acute pancreatitis as one of the etiologies of descending colonic strictures, especially in patients with no history of CD or TB, and a recent history of pancreatitis. Acute pancreatitis may involve the descending colon and can cause bowel obstruction even after a delay following the resolution of pancreatitis. Bowel resection may be warranted after the failure of medical and endoscopic therapy. Postoperative histology may assist in confirming the diagnosis.
